# Correction: Ding et al. MBD3 as a Potential Biomarker for Colon Cancer: Implications for Epithelial–Mesenchymal Transition (EMT) Pathways. *Cancers* 2023, *15*, 3185

**DOI:** 10.3390/cancers15215120

**Published:** 2023-10-24

**Authors:** Yuntao Ding, Huizhi Wang, Junqiang Liu, Han Jiang, Aihua Gong, Min Xu

**Affiliations:** 1 Department of Gastroenterology, Affiliated Hospital of Jiangsu University, Jiangsu University, Zhenjiang 212000, China; 2222113004@stmail.ujs.edu.cn (Y.D.); 2112013011@stmail.ujs.edu.cn (H.W.); 2221613035@stmail.ujs.edu.cn (J.L.); 2221913006@stmail.ujs.edu.cn (H.J.); 2 Hematological Disease Institute of Jiangsu University, Affiliated Hospital of Jiangsu University, Jiangsu University, Zhenjiang 212001, China

In the published paper [1], there was an error regarding the affiliation for Huizhi Wang. Yuntao Ding and Huizhi Wang contributed equally to this work and share co-first authorship, as stated in the Author Contributions section. The updated affiliation should include: †—These authors contributed equally to this work. The authors state that the scientific conclusions are unaffected. This correction was approved by the Academic Editor. The original publication has also been updated.
