# Supplementary material for: MBD3 as a Potential Biomarker for Colon Cancer: Implications for Epithelial-Mesenchymal Transition (EMT) Pathways
Source: Cancers (Basel). 2023 Jun 14;15(12):3185. doi: 10.3390/cancers15123185 (PMC10296356; doi:10.3390/cancers15123185)
Supplement: Supplementary file 1 [file cancers-15-03185-s001.zip › cancers-2424675-supplementary.pdf]

Supplementary Materials: MBD3 as a Potential Biomarker for Colon Cancer: Implications for Epithelial-Mesenchymal Transition (EMT) Pathways

Yuntao Ding, Huizhi Wang, Junqiang Liu [1],Han Jiang, Aihua Gong and Min Xu

A

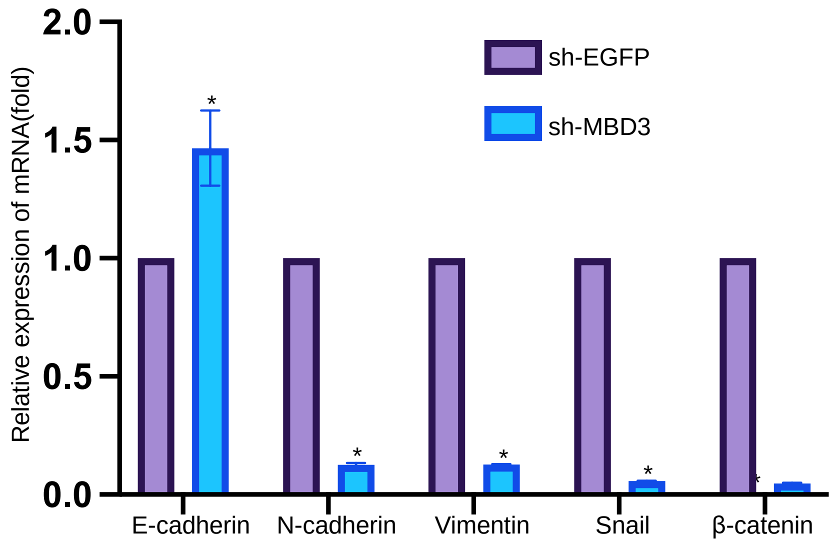

B

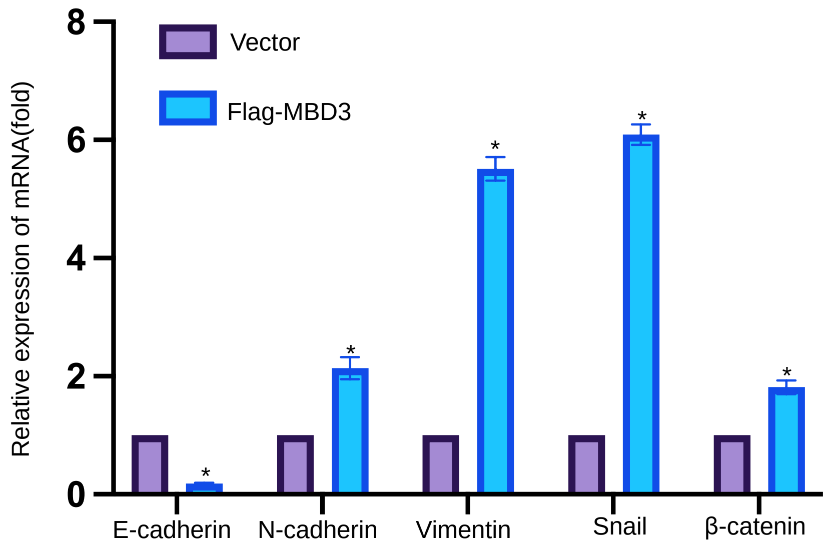

Figure S1. Differential expression of EMT-related molecules:(A) and (B):Differential expression of EMT-related molecules in SW620 cells of sh-MBD3 and CaCO2 cells of flag-MBD3.

Table S1. Association of full tumor names with abbreviations.

Cohort	Full Name
TCGA-ACC	Adrenocortical carcinoma
TCGA-BLCA	Bladder Urothelial Carcinoma
TCGA-BRCA	Breast invasive carcinoma
TCGA-CESC	Cervical squamous cell carcinoma and endocervical adenocarcinoma
TCGA-CHOL	Cholangiocarcinoma
TCGA-COAD	Colon adenocarcinoma
TCGA-COAD-READ	Colon adenocarcinoma/Rectum adenocarcinoma Esophageal carcinoma
TCGA-DLBC	Lymphoid Neoplasm Diffuse Large B-cell Lymphoma
TCGA-ESCA	Esophageal carcinoma
TCGA-FPPP	FFPE Pilot Phase II
TCGA-GBM	Glioblastoma multiforme
TCGA-GBMLGG	Glioma
TCGA-HNSC	Head and Neck squamous cell carcinoma
TCGA-KICH	Kidney Chromophobe
TCGA-KIPAN	Pan-kidney cohort (KICH+KIRC+KIRP)
TCGA-KIRC	Kidney renal clear cell carcinoma
TCGA-KIRP	Kidney renal papillary cell carcinoma
TCGA-LAML	Acute Myeloid Leukemia
TCGA-LGG	Brain Lower Grade Glioma
TCGA-LIHC	Liver hepatocellular carcinoma
TCGA-LUAD	Lung adenocarcinoma
TCGA-LUSC	Lung squamous cell carcinoma
TCGA-MESO	Mesothelioma
TCGA-OV	Ovarian serous cystadenocarcinoma
TCGA-PAAD	Pancreatic adenocarcinoma
TCGA-PCPG	Pheochromocytoma and Paraganglioma
TCGA-PRAD	Prostate adenocarcinoma
TCGA-READ	Rectum adenocarcinoma
TCGA-SARC	Sarcoma
TCGA-STAD	Stomach adenocarcinoma
TCGA-SKCM	Skin Cutaneous Melanoma
TCGA-STES	Stomach and Esophageal carcinoma
TCGA-TGCT	Testicular Germ Cell Tumors
TCGA-THCA	Thyroid carcinoma
TCGA-THYM	Thymoma
TCGA-UCEC	Uterine Corpus Endometrial Carcinoma
TCGA-UCS	Uterine Carcinosarcoma
TCGA-UVM	Uveal Melanoma
TARGET-OS	Osteosarcoma
TARGET-ALL	Acute Lymphoblastic Leukemia
TARGET-NB	Neuroblastoma
TARGET-WT	High-Risk Wilms Tumor

Table S2. Primer Oligonucleotides.

Name	Primer Sequence (5′-3′)
E-cadherin-F	5′-ATTTTTCCCTCGACACCCGAT-3′
E-cadherin-R	5′-TCCCAGGCGTAGACCAAGA-3′
β-catenin-F	5′-AAAGCGGCTGTTAGTCACTGG-3′
β-catenin-R	5′-CGAGTCATTGCATACTGTCCAT-3′.
Vimentin-F	5′-AGTCCACTGAGTACCGGAGAC-3′

Vimentin-R	5′-CATTTCACGCATCTGGCGTTC-3′
N-cadherin-F	5′- TCAGGCGTCTGTAGAGGCTT-3′
N-cadherin-R	5′-ATGCACATCCTTCGATAAGACTG-3′
Snail-F	5′-TCGGAAGCCTAACTACAGCGA-3′
Snail-R	5′-AGATGAGCATTGGCAGCGAG-3′
